# Supplementary material for: An open-source framework for large-scale, flexible evaluation of biomedical text mining systems
Source: J Biomed Discov Collab. 2008 Jan 29;3:1. doi: 10.1186/1747-5333-3-1 (PMC2276192; doi:10.1186/1747-5333-3-1)
Supplement: Additional file 1 — Evaluation platform source code. The additional file contains the software evaluation framework discussed in this paper. The most current release can be downloaded from SourceForge.net [1]. See the accompanying README file for instructions on installation and use. Also included in the distribution is a collection of UIMA wrappers for some commonly used BioNLP tools and annotated corpora. [file 1747-5333-3-1-S1.zip › bionlp_uima_repository-1.0.1/docs/api/src-html/edu/uchsc/ccp/uima/annotation/CCPSyntacticAnnotation.html]

```
001    
002    
003    /* First created by JCasGen Tue Aug 28 16:44:10 MDT 2007 */
004    package edu.uchsc.ccp.uima.annotation;
005    
006    import org.apache.uima.jcas.JCas;
007    import org.apache.uima.jcas.JCasRegistry;
008    import org.apache.uima.jcas.cas.TOP_Type;
009    
010    
011    
012    /** Super class for all syntactic annotations.
013     * Updated by JCasGen Tue Aug 28 16:44:10 MDT 2007
014     * XML source: /home/williamb/eclipse/RELEASED_SOURCE_CODE/descriptors/typeSystem/CCPTypeSystem.xml
015     * @generated */
016    public class CCPSyntacticAnnotation extends CCPTextAnnotation {
017      /** @generated
018       * @ordered 
019       */
020      public final static int typeIndexID = JCasRegistry.register(CCPSyntacticAnnotation.class);
021      /** @generated
022       * @ordered 
023       */
024      public final static int type = typeIndexID;
025      /** @generated  */
026      public              int getTypeIndexID() {return typeIndexID;}
027     
028      /** Never called.  Disable default constructor
029       * @generated */
030      protected CCPSyntacticAnnotation() {}
031        
032      /** Internal - constructor used by generator 
033       * @generated */
034      public CCPSyntacticAnnotation(int addr, TOP_Type type) {
035        super(addr, type);
036        readObject();
037      }
038      
039      /** @generated */
040      public CCPSyntacticAnnotation(JCas jcas) {
041        super(jcas);
042        readObject();   
043      } 
044    
045      /** @generated */  
046      public CCPSyntacticAnnotation(JCas jcas, int begin, int end) {
047        super(jcas);
048        setBegin(begin);
049        setEnd(end);
050        readObject();
051      }   
052    
053      /** <!-- begin-user-doc -->
054        * Write your own initialization here
055        * <!-- end-user-doc -->
056      @generated modifiable */
057      private void readObject() {}
058         
059    }
060    
061
```
